# Role of Programmed Cell Death Protein-1 and Lymphocyte Specific Protein Tyrosine Kinase in the Aryl Hydrocarbon Receptor- Mediated Impairment of the IgM Response in Human CD5^+^ Innate-Like B Cells

**DOI:** 10.3389/fimmu.2022.884203

**Published:** 2022-04-26

**Authors:** Jiajun Zhou, Lance K. Blevins, Robert B. Crawford, Norbert E. Kaminski

**Affiliations:** ^1^ Department of Microbiology & Molecular Genetics, Michigan State University, East Lansing, MI, United States; ^2^ Institute of Integrative Toxicology, Michigan State University, East Lansing, MI, United States; ^3^ Department of Pharmacology & Toxicology, Michigan State University, East Lansing, MI, United States; ^4^ Center for Research on Ingredient Safety, Michigan State University, East Lansing, MI, United States

**Keywords:** Lck, AhR (aryl hydrocarbon receptor), PD1 (programmed cell death protein 1), innate-like B cells, IFNgamma, IgM, TCDD

## Abstract

Innate-like B cells (ILBs) are a heterogeneous population B cells which participate in innate and adaptive immune responses. This diverse subset of B cells is characterized by the expression of CD5 and has been shown to secrete high levels of immunoglobulin M (IgM) in the absence of infection or vaccination. Further, CD5^+^ ILBs have been shown to express high basal levels of lymphocyte specific protein tyrosine kinase (LCK) and programmed cell death protein-1 (PD-1), which are particularly sensitive to stimulation by interferon gamma (IFNγ). Previous studies have demonstrated that activation of the aryl hydrocarbon receptor (AHR), a cytosolic ligand-activated transcription factor, results in suppressed IgM responses and is dependent on LCK. A recent study showed that CD5^+^ ILBs are particularly sensitive to AHR activation as evidenced by a significant suppression of the IgM response compared to CD5^-^ B cells, which were refractory. Therefore, the objective of this study was to further investigate the role of LCK and PD-1 signaling in AHR-mediated suppression of CD5^+^ ILBs. In addition, studies were conducted to establish whether IFNγ alters the levels of LCK and PD-1 in CD5^+^ ILBs. We found that AHR activation led to a significant upregulation of total LCK and PD-1 proteins in CD5^+^ ILBs, which correlated with suppression of IgM. Interestingly, treatment with recombinant IFNγ reduced LCK protein levels and reversed AHR-mediated IgM suppression in CD5^+^ ILBs in a similar manner as LCK inhibitors. Collectively, these results support a critical role for LCK and PD-1 in AHR-mediated suppression of the IgM response in human CD5^+^ ILBs.

## Introduction

Human B cells represent a highly diverse, adaptive immune cell population, which bridge adaptive and innate immunity. While murine B cells can be classified discretely as either B1 (innate) or B2 (conventional), no such classification scheme exists for human B cells. As the specific surface marker phenotype(s) of human-B1 B cells have yet to be defined and remain controversial in the field of human B cell biology, these cells can instead be loosely termed as Innate-like B cells (ILBs). ILBs comprise ~5-25% of circulating B cells and are a heterogeneous population of conventional and unconventional B cells, with most marked by expression of CD5 (1–5). As a mixed population of cells, human CD5^+^ ILBs include B1 B cells, marginal-zone B cells (MZ B cell), and B_Regulatory_ cells. Further, conventional immature B cells as well as follicular B cells (FO B cell) also express CD5 and are present in CD5^+^ B cell preparations (4, 5). CD5^+^ ILBs participate in innate and adaptive immune responses and contribute to the maintenance of a steady-state level of circulating natural IgM (nIgM). As a mixed cell population, CD5^+^ ILBs display a diverse range of immune functions upon activation, such as the secretion of IL-10, increasing production of nIgM, and secretion of proinflammatory mediators [i.e., interferon γ (IFNγ)] (6–9). Moreover, ILBs exhibit limited B cell receptor (BCR) rearrangement diversity and the association of CD5 with the BCR dampens antigen receptor signaling. Therefore, ILBs generally receive activation signals through CD40-CD40L interaction, toll-like receptors (TLR), and cytokines (10). nIgM plays a critical role in providing immunity against bacterial infections early in life during which the adaptive immune system is developing and late in life when the immune system is in decline (11). Despite being marked by their expression of CD5 in both humans and mice, distinct ILB subpopulations in humans are not well characterized when compared to murine ILBs (12).

As mentioned above, CD5^+^ ILB possess a wide range of effector functions, including regulatory mediators. Regulatory immune cells are marked by their ability to secrete inhibitory cytokines such as IL-10, but also by the expression of protein ligands which can signal through inhibitory receptors. Programmed cell death protein 1 (PD-1) is an inhibitory receptor which primarily functions to suppress immune responses (13). There are two well-characterized PD-1 ligands, PDL1 and the higher affinity PDL2. PDL1 is expressed by many immune and non-immune cells, but PDL2 expression is primarily restricted to antigen presenting and regulatory cells (14, 15). The binding of ligands to PD-1 initiates inhibitory signaling cascades that suppress immune responses on PD-1 expressing target cells. Interestingly, high basal expression of PD-1 has been reported in CD5^+^ ILBs (16, 17). PD-1 signaling involves the phosphorylation of the immunoreceptor tyrosine switch motifs (ITSMs) and immunoreceptor tyrosine inhibitory motifs (ITIMs) (14). Phosphorylated PD-1 then recruits Src homology 2 (SH2) domain containing phosphatases such as SHP-1, SHP-2, and others, which can then negatively regulate immune activation and antigen receptor signaling in T and B cells (18, 19).

Lymphocyte specific protein tyrosine kinase (LCK) is a critical signaling molecule associated with T cell receptor (TCR) and PD-1 signaling (20, 21). Upon TCR engagement, phosphorylation at the immunoreceptor tyrosine-based activation motif (ITAM) transfers a phosphate group to LCK (22). Activation of LCK is critical for Ca^2+^ mobilization (23) and the activation of MAP kinase and NFAT pathways following TCR engagement (24). The role of LCK has been well-characterized in T cells; however, little is known concerning the role of LCK in B cells. Studies have identified elevated LCK expression within CD5 expressing B cells (11, 25) and the increase of LCK has been implicated as a biomarker for the progression of CLL patients (11, 26). A recent publication reported that human peripheral CD5^+^ B cells characteristically express high levels of LCK (27). LCK has also been reported to be one of the primary kinases responsible for the phosphorylation of PD-1 cytoplasmic tail ITIM sequences in T cells (18).

In contrast to inhibitory effector functions, ILBs have also been described as promoting inflammation *via* the release of proinflammatory mediators such as interferon γ (IFNγ). IFNγ is a well characterized cytokine that plays a diverse role in cellular programs through transcriptional regulation and promotes the clearance of pathogens. IFNγ is primarily produced by lymphocytes and mucosal epithelial cells to promote innate and cell-mediated immunity (28). IFNγ secretion by lymphocytes and mucosal epithelial cells is important in the early stages of host defense against infections, whereas T cells are the major source of IFNγ in the adaptive immune response (29–31). Importantly, ILBs have also been reported to produce and respond to IFNγ (32, 33). IFNγ has also been shown to modulate cell cycle, cell growth and apoptosis (34, 35). Binding of IFNγ to its receptor, which consists of IFNGR1 and IFNGR2, activates the Jak-Stat pathway (36, 37).

Finally, aryl hydrocarbon receptor (AHR) is a cytoplasmic ligand activated transcription factor involved in the regulation of cell functions and xenobiotic sensing (38–42). AHR also plays a biological role in immune modulation and differentiation, especially in the expansion, maturation and differentiation of B cells (43–47). High affinity, anthropogenic ligands like 2,3,7,8-tetrachlorodibenzo-*p*-dioxin (TCDD) have been widely used to study the physiological role of the AHR in B cells. B cells are a sensitive target for AHR activation as evidenced by significant impairment of B cell lineage commitment and suppression of humoral immune responses (48–50). AHR activation has also been demonstrated to decrease critical cellular transcription factors and signaling molecules involved in B cell differentiation into antibody producing plasma cells in humans (51). While the underlying mechanism by which the AHR modulates antibody responses in B cells is still largely unknown, recent studies have demonstrated AHR activation induced LCK expression in human primary B cells and LCK is critical for optimal IgM secretion (52). Indeed, a recent report from Blevins and colleagues demonstrated that CD5^+^ ILB were preferentially sensitive to AHR-mediated suppression of IgM secretion (17). Moreover, AHR-mediated induction of LCK protein expression was limited to CD5^+^ ILB, further implicating a role for LCK in AHR-mediated suppression of IgM secretion (17). Consequently, Blevins et al. also found that TCDD-treatment of human CD5^+^ ILB significantly enhanced PD-1 protein expression, providing a putative mechanism for AHR-mediated IgM suppression (17). Curiously, it has also been reported that treatment with exogenous IFNγ blocked AHR-mediated suppression of the IgM response in mouse splenocytes as well as human B cells (53, 54). Currently no published studies have demonstrated a direct role of AHR and IFNγ on PD-1 and LCK regulation; however, it has been previously demonstrated that IFNγ, as well as other cytokines, can directly regulate the expression of both PD-1 and PDL1 (55–57).

In this study, enriched CD5^+^ and CD5^-^ B cells were utilized to investigate the role of AHR activation in the context of CD5^+^ ILB function. Here we show a significant increase of LCK and decrease in the IgM response with AHR activation in CD5^+^ ILBs. However there was no effect on LCK inhibitory phosphorylation. Treatment of CD5^+^ ILBs with either AHR- or LCK-specific inhibitors similarly blocked AHR-mediated suppression of the IgM response. We report for the first time that PD-1 is functionally competent in human CD5^+^ ILB as treatment with soluble ligands suppressed IgM secretion and PD-1 function was blocked by LCK inhibitor treatment. Similarly, treatment with an anti-PD-1 blocking antibody also prevented AHR-mediated suppression of the IgM response in CD5^+^ ILBs. Finally, we report for the first time that TCDD-mediated AHR activation significantly induced PDL2, but not PDL1, on human CD5^+^ ILB. Collectively, these results suggest LCK, PD-1, and IFNγ play a critical role in the regulation of IgM responses in the context of AHR activation in CD5^+^ ILBs.

## Materials and Methods

### Chemicals and Reagents

99.1% pure TCDD dissolved in dimethylsulfoxide (DMSO) was from AccuStandard Inc (New Haven, Connecticut). DMSO was from Sigma-Aldrich (St. Louis, Missouri) and was used to dilute the TCDD. DMSO (0.02% final concentration in culture) was used in all treatments. LCK inhibitor (CAS213743-31-8) was from Sigma-Aldrich. AHR antagonist, CH223191 (≥98.0% purity), dissolved in DMSO was purchased from Tocris (Bristol, United Kingdom). Human recombinant IFN-gamma (IFNγ) protein was from Biolegend (San Diego, California). PD-1 blocking antibody (S228P) and PDL1 blocking antibody [Anti-hPD-L1-hIgG1 (N298A)] were from *In vivo*Gen (San Diego, California). PDL2 blocking antibody (clone 24F.10C12) was from Millipore Sigma (Burlington, Massachusetts).

*Human leukocyte packs and human B cell purification*: peripheral blood mononuclear cells (PBMCs) collected from anonymous platelet donors were obtained from Gulf Coast Regional Laboratories (Houston, Texas). All human leukocyte packs were tested to be negative for human immunodeficiency virus (HIV), hepatitis B virus (HBV), hepatitis C virus (HCV) and human T-lymphocyte virus (HTLV). For each experiment, blood packs were diluted with HBSS and overlaid on Ficoll-Paque Plus density gradient (GE Healthcare, Piscataway, New Jersey) and centrifuged at 1300 x g for 25 min with low acceleration and brake. The PMBCs were isolated after centrifugation, washed, counted and subjected to magnetic isolation that enriched CD19^+^CD27^-^ naïve human B cells (greater than 95% purity). This negative selection was conducted using the MojoSort™ human naïve B cell isolation kit (Biolegend, San Diego) per manufacturer’s instructions. After the first isolation for total B cells, a second isolation was conducted to enrich CD5^+^ ILBs. In brief, 10 μL of biotin anti-human CD5 antibodies per 10^6^ cells was incubated with naïve B cells for 15 min on ice, followed by addition of 10 μL of anti-biotin microbeads per 10^6^ cells for an additional 15 min. Details for enrichment of CD5^+^ B cells can be found in (17). Purified CD5^+^ ILBs at the concentration of 0.25x10^6^ cells/mL were then treated with either 0.02% DMSO (Veh) or 10 nM TCDD. The treated ILBs were then activated by soluble human CD40 ligand (100 ng/mL) (Enzo, Farmingdale, New York) and supplied with recombinant human cytokines IL-2 (1 ng/ml) (Roche Applied Science, Indianapolis, Indiana) and IL-21 (100 ng/mL) (R&D system, Minnesota) for a total of 7 days.

### Enzyme-Linked Immunospot (ELIspot) Assay

The number of IgM-secreting cells was quantified by ELISPOT. Multiscreen 96-well filter plates (Millipore, Billerica, Massachusetts) were coated with anti-human IgM antibody (5 μg/ml) (Sigma Aldrich, St. Louis, MO) overnight and subsequently blocked with 5% bovine serum albumin (Sigma Aldrich, St. Louis, MO) for 2 h. Cells were washed with RPMI 1640 twice, resuspended in RPMI 1640 containing 10% bovine calf serum (Thermo Scientific, Lafayette, Colorado) and incubated on the primary antibody-coated plates overnight at 37°C with 5% CO_2_. Biotin-conjugated anti-human IgM antibody (Sigma Aldrich, St. Louis, MO) and streptavidin horseradish peroxidase (HRP) (Sigma Aldrich, St. Louis, MO) were added for one-hour and incubated at 37°C with 5% CO_2_. All incubations were followed by three washes with phosphate-buffered saline (pH 7.4) containing 0.1% Tween-20 (Sigma Aldrich, St. Louis, MO) and three washes with nanopure water. The spots were developed with an aminoethylcarbazole staining kit (Sigma Aldrich, St. Louis, MO). The number of spots per well between 0.0001mm^2^ and 9.6372mm^2^ were quantified *via* the Immunospot Software (Cellular Technology, Ltd, Shaker Heights, Ohio) and normalized to the number of viable cells collected from each well.

### Enzyme-Linked Immunosorbent (ELISA) Assay

The quantity of IgM antibodies secreted into the culture supernatants was determined by sandwich ELISA. Briefly, Immulon 4 HBX 96-well microtiter plates (VWR International, Radnor, Pennsylvania) were coated with anti-human IgM antibody (1 μg/ml; Sigma Aldrich, St. Louis, MO) overnight. Supernatants collected from human B cell cultures were incubated over primary antibody-coated plates for 90 min at 37°C with 5% CO_2_ and followed by overlaying an anti-human IgM-HRP conjugate antibody (Sigma Aldrich, St. Louis, MO). Each of the incubations was followed by washes with phosphate-buffered saline (pH 7.4) containing 0.05% Tween-20 (Sigma Aldrich, St. Louis, MO) and nanopure water. 2,2’-Azino-bis (3-ethylbenzothiazoline-6-sulphonic acid) (ABTS, Roche Diagnostics) was then added as a colorimetric substrate for HRP. The rate of colorimetric change was quantified with a Synergy HT microplate reader (BioTek, Winooski, Vermont) at 405 nm for 1 h. The concentration of IgM in supernatants was calculated based on a standard curve created in each plate.

### Flow Cytometry

Antibodies used for flow cytometry were as follows: Alexa Flour 647 anti-human LCK (LCK-01), APC anti-human CD5 (UCHT2), Biotin anti-human CD5 (UCHT2), APC anti-human CD279 (PD-1, EH122H7), PE CD273 (PDL2, 24F.10C12), APC CD273 (PDL2, 24F.10C12), and Per/Cy5.5 CD274 (PD-L1, 29E.2A3) (all from Biolegend). Alexa Fluor 488 conjugated phosphorylated LCK tyrosine 505 (pLCK Y505) (clone 4/LCK-Y505) was purchased from BD Biosciences. For flow cytometry staining, approximately 0.25x10^6^ cells were harvested at the indicated time points and viable cells were identified by Fixable Live/Dead Near-IR dye (Life Technologies) per manufacturer’s instructions prior to cell surface or intracellular staining. Surface Fc receptors were blocked using human AB serum before staining for surface and intracellular proteins. For surface staining, cells were resuspended in FACS buffer (1x phosphate-buffered saline, 1% bovine serum albumin (58) (San Diego, California) and 0.1% sodium azide, pH: 7.6) in the presence of 20% human AB serum. Antibodies were added per manufacturer’s specified concentrations, incubated at 4°C for 15 min and then fixed by incubation in the BD Cytofix fixation buffer (BD Biosciences) for 10 min. For intracellular protein staining, cells that were previously fixed after surface staining were permeabilized with 1X BD PermWash buffer (BD Biosciences) for 30 min and incubated with antibodies for 30 min. In all cases, cells were analyzed by a BD FACSCanto II using FACS Diva software (BD Biosciences) and subsequently analyzed using FlowJo (Version 10, Treestar Software Ashland, Oregon). Unless otherwise stated, cells were first gated on singlets, then live (as determined by Live/Dead dye), and followed by gating on lymphocyte populations. Gates were drawn based on the unstimulated cells (resting human B cells, without CD40L and cytokine activation) or unstained cells as appropriate.

### Statistical Analysis

Linear regression was used in the correlation study. Student’s t-test was used to compare Veh control to TCDD treatment group. For multiple comparisons, unless otherwise stated in the figure legend, one-way ANOVA followed by Fisher’s LSD *post hoc* test or two-way ANOVA followed by Fisher’s LSD *post hoc* test was used. Significant differences from Veh control were indicated by * *p* < 0.05, ** *p* < 0.01, *** *p* < 0.001. Significant differences from TCDD-treated control were indicated by # *p* < 0.05 and ## *p* < 0.01. The error-bars represent standard deviation.

## Results

### AHR-Mediated Increase in the Percentage of LCK^+^ Cells and Suppression of the IgM Response in CD5^+^ ILBs

**Figure 1:**
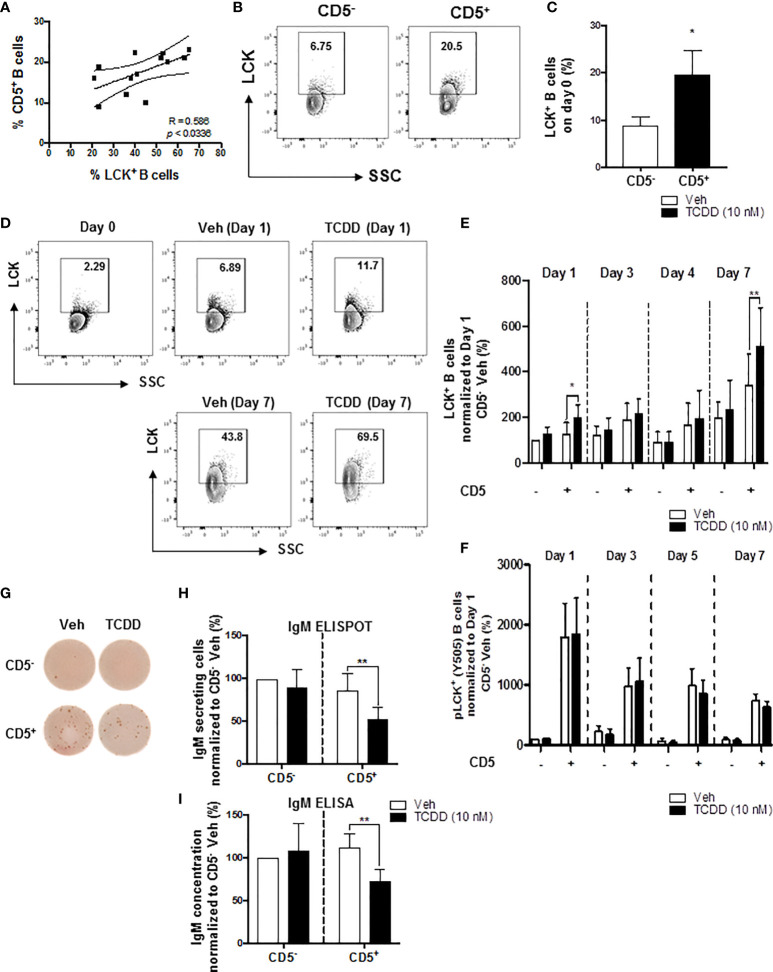
TCDD-mediated increase in the percentage of LCK^+^ human CD5^+^ ILBs and the suppression of the IgM response. Human CD5^+/-^ B cells were activated with CD40L, IL-21, and IL-2 and treated with Veh (0.02% DMSO), or TCDD (10 nM) on day 0 and cultured for 7 days. Cells and culture supernatants were collected and assessed for LCK and LCK Y505 phosphorylation by flow cytometry and IgM secretion *via* ELISA and ELIspot. **(A)** Correlation of percent CD5^+^ B cells and percent LCK^+^ B cells; **(B)** Representative flow cytometry plots of CD5^+^ and LCK^+^ cells; **(C)** Percentage of LCK^+^ B cells within the CD5^+/-^ populations on day 0; **(D)** Representative flow cytometry plots of LCK^+^ cells within CD5^+/-^ populations; **(E)** Percentage of LCK^+^ B cells within CD5^+/-^ populations on day 1, 3, 4 and 7 with Veh or TCDD treatment; **(F)** Percentage of pLCK (Y505)^+^ B cells within CD5^+/-^ populations on day 1, 3, 5 and 7 with Veh or TCDD treatment; **(G)** Representative ELIspot wells showing IgM secreting cells measured within CD5^+/-^ populations with Veh or TCDD (10 nM) treatment on day 7; **(H)** Number of IgM secreting cells; and **(I)** IgM concentration from culture supernatants from CD5^+/-^ B cells treated with Veh or TCDD. Determinations were made using B cells from 6 human donors (N = 6). For **(E, F)**, data were normalized to the CD5^-^ Veh on day 1. For **(H, I)**, data were normalized to CD5^-^ Veh. Significant differences are indicated by *p < 0.05 and **p < 0.01 (Student’s T test or two-way ANOVA following with Fisher’s LSD *post hoc* test).

We have previously shown that AHR activation by TCDD concomitantly upregulated LCK and suppressed the IgM response in CD40-ligand activated total human B cells (52). Moreover, the suppression of the IgM response by AHR activation was reversed by treatment of B cells with LCK inhibitors (52). More recently, studies by Blevins et al. demonstrated that CD5^+^ ILBs showed preferential sensitivity to AHR-mediated impairment of the IgM response (17). In the current study, we show a positive correlation between the percentage of circulating CD5^+^ ILBs in any given donor and the percentage of LCK^+^ CD5^+^ ILBs (**Figure 1A**). Initially, after enrichment, the level of total LCK protein was found to be 3-times greater in CD5^+^ B cells when compared to CD5^-^ B cells on day 0, with an average of ~20% of CD5^+^ B cells staining positive for LCK protein versus ~7% in CD5^-^ B cells (**Figures 1B, C**). When CD5^+^ B cells were activated with CD40-ligand, TCDD-mediated AHR activation increased the total LCK levels when compared to the 24 hour vehicle treated CD5^-^ B cells as a comparator, with the most marked increases taking place on days 1 and 7 (**Figures 1D, E**). As the kinase activity of LCK is regulated by a dominant, inhibitory phosphorylation on residue tyrosine 505 (59), we also assessed the activity level of LCK in TCDD treated CD5^+^ and CD5^-^ B cells. In contrast to total LCK, TCDD-mediated AHR activation did not appear to modulate pLCK Y505 phosphorylation in either CD5^+^ or CD5^-^ B cells suggesting AHR regulates LCK expression but not kinase activity (**Figure 1F**). In addition, we verified that TCDD-mediated AHR activation induced suppression of the IgM response in CD5^+^ ILBs. As shown in **Figure 1G**, CD5^+^ B cells secreted more IgM compared to CD5^-^ B cells when activated as described. Further, CD5^+^ B cells exhibited marked IgM suppression which occurred concordantly with LCK upregulation (**Figures 1H, I**). Conversely, CD5^-^ B cells were refractory to IgM suppression by AHR activation as TCDD treatment did not significantly affect IgM secretion (**Figures 1G-I**). However, there was a slight trend toward reduced IgM^+^ spots when IgM was enumerated *via* ELISPOT in CD5^-^ B cells treated with TCDD that was not apparent using ELISA (**Figures 1H, I**).

### AHR Antagonist Blocked TCDD-Induced Upregulation of LCK and the Suppression of the IgM Response in CD5^+^ ILBs

**Figure 2:**
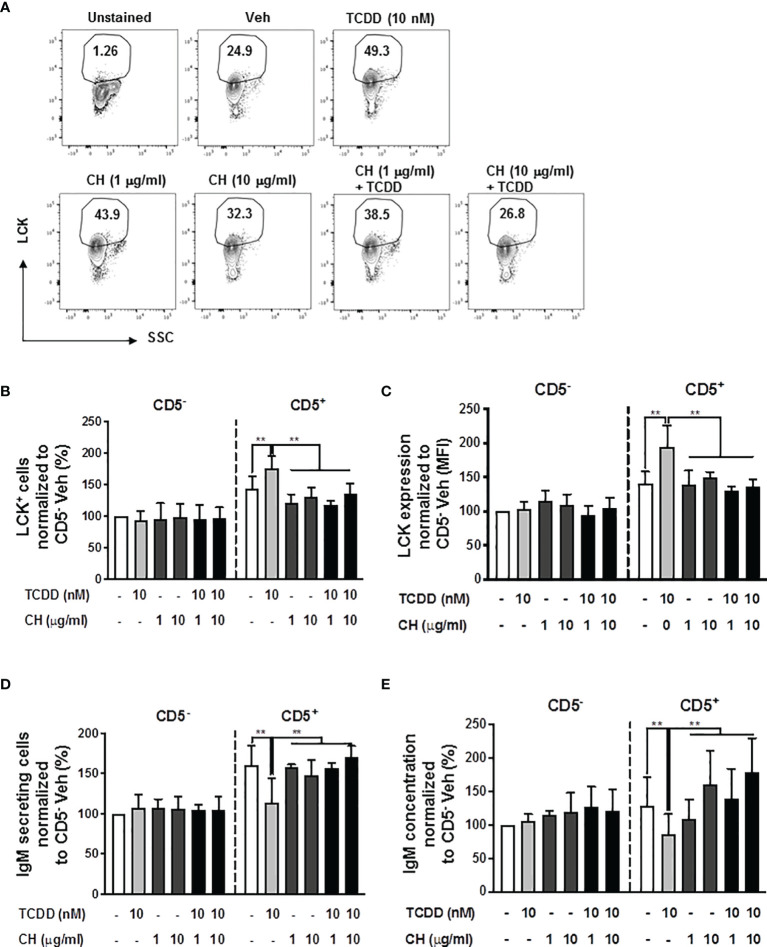
The TCDD-mediated upregulation of LCK and suppression of the IgM response in CD5^+^ ILBs is blocked by AHR antagonist treatment. Human CD5^+/-^ B cells were pre-treated with 1 or 10 μg/ml of AHR antagonist (CH-223191 abbreviated as CH), for 30 minutes and then followed by activation and treatment with Veh (0.02% DMSO) or TCDD (10 nM) on day 0 and cultured for 7 days as previously described. **(A)** Representative flow cytometry plots of CD5^+^ ILBs with or without AHR agonist and/or antagonist treatment; **(B)** Percentage of LCK^+^ cells; **(C)** Mean florescence intensity (MFI) of LCK in CD5^+/-^ populations; **(D)** Number of IgM secreting cells; and **(E)** IgM concentration within CD5^+/-^ populations with Veh or TCDD treatment. Determinations were made using B cells from 6 human donors (N = 6) from 2 independent experiments. Data were normalized to the CD5^-^ Veh control group. Significant differences are indicated by **p < 0.01 (two-way ANOVA following with Fisher’s LSD *post hoc* test).

To directly test the role of the AHR in mediating the induction of LCK and the suppression of IgM responses in CD5^+^ ILBs, the AHR antagonist, CH-223191 (CH), was employed. Briefly, human B cells were enriched into CD5^+^ and CD5^-^ populations and activated as previously described. Enriched cells were then treated with either DMSO or TCDD in the presence or absence of CH. Addition of AHR antagonist at either 1 or 10 μg/mL or in combination with TCDD treatment both resulted in a reduction in the frequency of total LCK^+^ cells when compared to TCDD treated CD5^+^ ILBs (**Figures 2A, B**). Similarly, AHR antagonist treatment, alone or with TCDD, also decreased the levels of LCK on a per cell basis when compared to TCDD treated CD5^+^ ILB (**Figure 2C**). Further, when we quantified IgM secretion in AHR antagonist and TCDD treated B cells, antagonist treatment alone had no effect on the IgM response by CD5^+^ or CD5^-^ B cells (**Figures 2D, E**). However, when AHR antagonist was in combination with 10 nM TCDD, IgM secretion was restored when compared to TCDD treatment alone (**Figures 2D, E**). These results demonstrate a direct role for AHR activation in the enhancement of LCK expression and subsequent suppression of IgM secretion in CD5^+^ ILBs.

### Inhibition of LCK Reverses AHR-Mediated Suppression of IgM Responses in CD5^+^ ILBs

**Figure 3:**
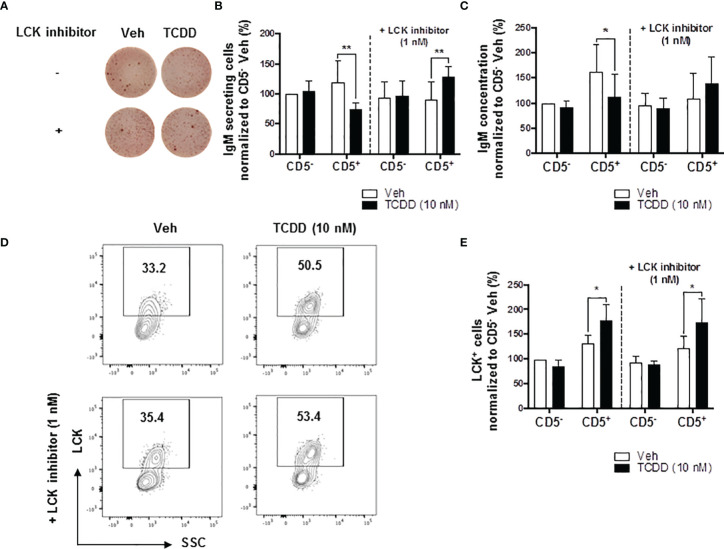
LCK inhibitor treatment reversed the AHR-mediated suppression of the IgM response in CD5^+^ ILBs. Human CD5^+/-^ B cells were activated and treated with Veh or TCDD on day 0 and cultured for 7 days as described previously. LCK inhibitor (RK24466) was added on day 5 to the cell culture. **(A)** Representative ELIspot wells showing IgM secreting cells measured within CD5^+/-^ B populations on day 7; **(B)** Averaged number of IgM secreting cells and; **(C)** Averaged IgM concentration within culture supernatants collected from Veh or TCDD-treated CD5+/- cultures with and without LCK inhibitor; **(D)** Representative flow cytometry plots of LCK^+^ CD5^+^ ILBs with or without LCK inhibitor treatment; and **(E)** Averaged percentage of LCK^+^ cells within CD5^+/-^ populations with or without LCK inhibitor treatment. Determinations were made using B cells from 6 human donors (N = 6) from two independent experiments. Data presented in the figures were normalized to the CD5^-^ Veh without LCK inhibitor treatment. Significant differences are indicated by *p < 0.05 and **p < 0.01 (two-way ANOVA following with Fisher’s LSD *post hoc* test).

Building on the observations above, we directly tested the role of elevated LCK protein in the AHR-mediated inhibition of IgM secretion by CD5^+^ B cells. Previous studies have shown that LCK inhibitor (RK24466) treatment was able to reverse AHR-mediated suppression of IgM secretion in total B cells (52). Here we extend this observation by demonstrating that treatment with RK24466 (1 nM) restored IgM secretion by CD5^+^ B cells in the presence of TCDD-mediated AHR activation. As shown in **Figures 3A–C**, treatment of CD5^+^ ILB with LCK inhibitor alone appeared to reduce IgM secretion when compared to vehicle treated CD5^+^ ILB, which was in agreement with previously published observations in total CD19^+^ B cells (52). However, when LCK inhibitor was added in combination with 10 nM TCDD, LCK inhibitor blocked TCDD-mediated suppression of the IgM response restoring IgM secretion to levels similar to those in vehicle treated CD5^+^ B cells (**Figures 3B, C**). However, RK24466 treatment did not affect the IgM response by CD5^-^ B cells, with or without TCDD treatment (**Figures 3B, C**). Next the effect of LCK inhibition on the expression of total LCK protein was determined. Consistent with previous observations, the percentage of LCK^+^ cells continued to increase with AHR activation despite the presence of LCK inhibitor (**Figures 3D, E**). Taken together, these data suggest a putative role for LCK kinase activity in the AHR-mediated suppression of IgM secretion as its inhibition restored the IgM response in the absence of altering LCK protein levels.

### TCDD-Mediated AHR Activation Significantly Enhances PDL2, but Not PDL1, Expression on CD5^+^ Innate-Like B Cells

**Figure 4:**
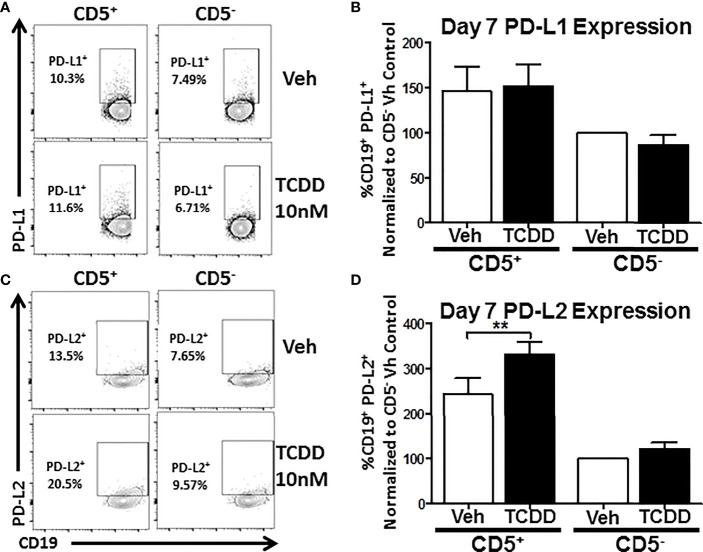
TCDD treatment of human CD5^+^ B cells significantly increases the frequency of PD-L2 cell surface protein positive cells. Human CD5^+^ and CD5^-^ B cells were isolated, activated, and treated with TCDD (10nM) for 7 days as previously described. After the culture period, cells were collected and surface stained with anti-PD-L1 and PD-L2 antibodies and cell surface protein expression was quantified by flow cytometry. Representative flow plots for Veh and TCDD treated CD5^+/-^ B cells are shown in panels **(A, C)**. PD-L1^+^ and PD-L2^+^ cells were identified in the lymphocyte, singlet gate by gating on live CD19^+^ cells. The frequency of PD-L1 and PD-L2 positive cells was normalized to CD5^-^ Veh control. Averaged data from 2 independent experiments assessing a total of 6 human donors are shown in panel **(B)** (PD-L1) and **(D)** (PD-L2). Significance was determined by a repeated measures two-way ANOVA with a Dunnett’s posttest. **p<0.01.

As previous work had found a putative role for AHR in PD-1 protein expression (17), we wanted to ascertain whether its ligands, PDL1 and PDL2 were similarly affected by TCDD-mediated AHR activation. To test this, human naïve B cells were isolated from PBMC as previously described and further enriched for expression of CD5. CD5^+^ and CD5^-^ B cells were then activated and treated with 10 nM TCDD as described. After 7 days in culture, cells were collected and stained for CD19, PDL1, and PDL2. As shown in **Figure 4**, panels A and C, we detected PDL1 and PDL2 protein expression on both CD5^+^ and CD5^-^ B cells with a higher frequency of CD5^+^ B cells staining positive (**Figures 4B, D**). When we compared the frequency of expression of PDL1 in TCDD-treated CD5^+/-^ B cells, we found no significant change in the percentage of cells expressing PDL1 in either cell population (**Figure 4B**). However, when we compared the percentage of PDL2 positive cells in CD5^+^ B cells to its vehicle control, we observed a 42% increase in the frequency of PDL2 positive cells (**Figure 4D**). TCDD treatment of CD5^-^ B cells had no effect on the percentage of PDL2 positive cells (**Figure 4D**). Together these data suggest that TCDD-mediated AHR activation positively regulates the expression of PDL2, but not PDL1. As PDL2 is the higher affinity ligand for PD-1, this further supports a putative role for AHR activation in promoting PD-1-mediated immune suppression.

### The Kinetic Profile of Activation-Induced Expression of PDL1 and PDL2 on the Cell Surface of CD5^+^ and CD5^-^ Human B Cells

**Figure 5:**
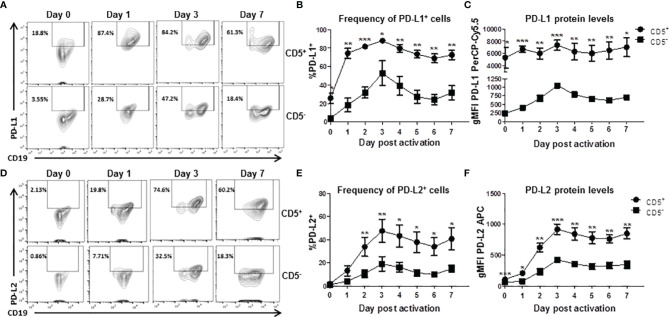
Kinetics of PD-L1 and PD-L2 cell surface protein expression by activated human CD5^+^ ILB. Human CD5^+/-^ B cells were activated and cultured for 7 days as described previously and cells were collected daily and assessed for surface expression of either PD-L1 or PD-L2 by flow cytometry. **(A)** Representative PD-L1 flow plots at days 0, 1, 3, and 7; **(B)** Averaged frequency of PD-L1^+^ cells; **(C)** Averaged PD-L1 PerCP-Cy5.5 geometric mean fluoresence intensity; **(D)** Representative PD-L2 flow plots at days 0, 1, 3, and 7; **(E)** Averaged frequency of PD-L2^+^ cells; and **(F)** Averaged PD-L2 APC geometric mean fluoresence intensity. Determinations were made using B cells from 6 human donors (N = 6) across 3 independent experiments. Signficant differences between CD5^+/-^ B cells were determined at each time point using a paired, two way t-test. Significant differences are indicated by *p < 0.05, **p < 0.01, and ***p < 0.001.

While we observed significant enhancement of TCDD-mediated AHR activation on the frequency of CD5^+^ B cells expressing PDL2, those measurements were performed on day 7 post activation. As such, we wanted to understand the kinetics of expression of both PDL1 and PDL2 in CD5^+^ as well as CD5^-^ B cells. Developing a kinetic profile of PDL1/PDL2 protein expression will help determine the optimal time to examine ligand expression as well as suggest putative timepoints for experimental intervention. As such, CD5^+^ and CD5^-^ B cells were activated as previously described and collected at 24 hour intervals to determine PDL1 and PDL2 protein expression by flow cytometry. As shown in **Figures 5A, D**, both CD5^+^ and CD5^-^ B cells respond to activation stimuli by increasing the percentage of PDL1/PDL2 positive cells. Indeed, a significant percentage of CD5^+^ B cells expressed PDL1 directly *ex vivo* when compared to CD5^-^ B cells. However, by as soon as 24 hours post activation, nearly 80% of CD5^+^ B cells expressed PDL1 compared to 20% of CD5^-^ B cells (**Figure 5B**). A higher frequency of CD5^+^ B cells expressed more PDL1 protein compared to CD5^-^ B cells at each time point tested (**Figures 5B, C**).

Similarly, CD5^+^ B cells expressed more PDL2 protein at each tested timepoint (**Figure 5F**) but the frequency of PDL2 positive CD5^+^ B cells did not differ compared to CD5^-^ B cells in the first 48 hours of activation (**Figure 5E**). This would suggest the kinetics of PDL2 expression are delayed in CD5^+^ B cells compared to PDL1, which is expressed at higher levels under basal conditions (**Figures 5B, C**). These results show that CD5^+^ B cells express significant levels of PDL1 *ex vivo* and in relation to CD5^-^ B cells, with activation further increasing PDL1 expression (**Figures 5B, C**). PDL2 appears to be regulated in a similar manner; however, there is little relative expression of PDL2 before activation (**Figures 5E, F**). Interestingly, day 3 post activation was the peak of expression for both PDL1 and PDL2 in both CD5^+^ and CD5^-^ B cells.

### Treatment With Soluble PD-1 Ligands (PDL1 and PDL2) Suppressed the IgM Response in CD5^+^ ILBs

**Figure 6:**
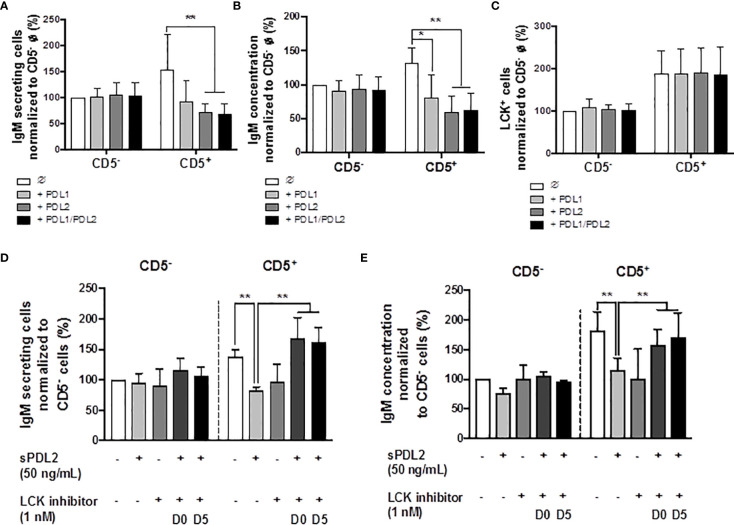
Treatment with soluble PD-1 ligands (PDL1 and PDL2) suppressed the IgM response and LCK inhibitor treatment blocked PD-1-mediated IgM suppression in CD5^+^ ILBs. Human CD5^+/-^ B cells were activated as previously described and treated with soluble PD-L1, PD-L2 or the combination of both on day 0 and cultured for 7 days. LCK inhibitor (RK24466) was provided on day 0 or day 5 to the cell culture. **(A)** Averaged ELIspot data showing the number of IgM secreting cells; **(B)** Averaged IgM concentration from culture supernatants; **(C)** Averaged frequency of LCK^+^ cells within CD5^+/-^ populations on day 7; **(D)** Averaged number of IgM secreting cells; and **(E)** Averaged IgM concentration from CD5^+/-^ culture supernatants with PDL2 and LCK inhibitor treatments. Determinations were made using B cells from 6 human donors (N = 6) across two independent experiments. For **(A–C)**, data presented in the figure were normalized to CD5^-^ B cells without PD-1 ligand treatment. For **(D, E)**, results were normalized to CD5^−^ B cells without PDL2 or LCK inhibitor treatment. Significant differences are indicated by *p < 0.05 and **p < 0.01 (two-way ANOVA following with Fisher’s LSD *post hoc* test).

Previous studies have identified higher basal expression of PD-1, and its ligands, by CD5^+^ ILBs, compared to CD5^-^ B cells (17). Currently, there are no reports describing the role of PD-1 signaling in the secretion of IgM by human B cells; however, there is limited evidence that PD-1 signaling suppresses Ig secretion in mice (60, 61). Therefore, we further investigated the involvement of PD-1 and its ligands in modulating IgM responses of CD5^+^ ILBs using soluble PD-1 ligands (sPDL1 and sPDL2). The ligand concentrations used in the current studies were chosen such that they would provide approximately similar stimulatory signals based on their binding affinity for PD-1 (sPDL1: 1 μg/mL and sPDL2: 50 ng/mL). Treatment with sPDL1, sPDL2 or the combination of both ligands produced significant suppression of the IgM response by CD5^+^ ILBs (**Figures 6A, B**); however, greater suppression was observed with sPDL2 or in combination with sPDL1 (**Figures 6A, B**). In addition, PD-1 ligand treatment did not change the percentage of LCK^+^ CD5^+^ ILBs and CD5^-^ B cells (**Figure 6C**).

### LCK Inhibitor Reversed the PD1-Mediated Suppression of the IgM Response in CD5^+^ ILBs

LCK has been shown to have an important role in phosphorylating the ITSM domain on PD-1 for the docking of SHP-1 or SHP-2 (62). Based on previous observations, the level of LCK increased significantly with AHR activation in CD5^+^ ILBs. Therefore, studies were conducted to further understanding the involvement of LCK in PD-1 signaling by using a specific LCK inhibitor (RK24466) (1 nM) in combination with PDL2 (50 ng/mL) treatment in CD5^+/-^ B cells. PDL2 was selected based on the previous observation that PDL2 treatment was more potent in suppressing the IgM response in CD5^+^ B cells (**Figures 6A, B**). In this series of experiments, the LCK inhibitor was added directly to the cells on day 0 (D0) or day 5 (D5), post activation. LCK inhibitor treatment at both time points significantly reversed the PD-1-mediated IgM suppression in only CD5^+^ ILBs (**Figures 6D, E**).

### Treatment With PDL2 Did Not Further Suppress the IgM Response Than AHR Activation Alone in CD5^+^ ILBs

**Figure 7:**
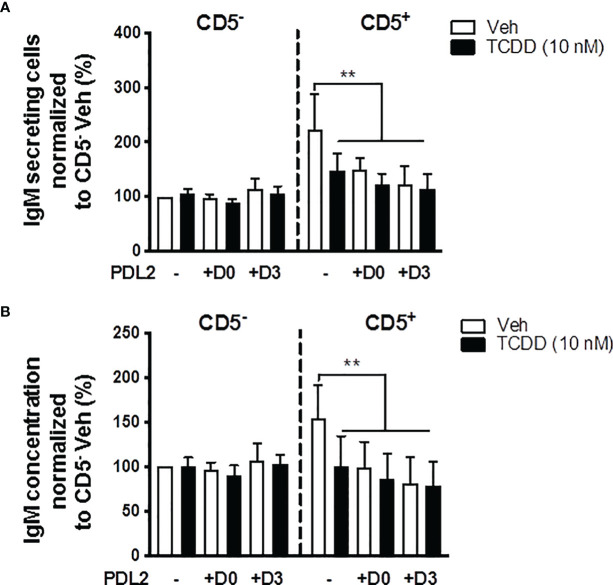
Soluble PDL2 treatment did not further suppress the IgM response compared to AHR activation alone in CD5^+^ ILBs. Human CD5^+/-^ B cells activated as described were treated with Veh or TCDD in the presence or absence of soluble PD-L2. Soluble PDL2 was added to the culture on day 0 or day 3. **(A)** Averaged number of IgM secreting cells; and **(B)** Averaged IgM concentrations from CD5^+/-^ culture supernatants on day 7. Determinations were made using B cells from 6 human donors (N = 6). Data presented in the figure were normalized to Veh treated CD5^-^ B cells without PDL2 treatment. Significant differences are indicated by **p < 0.01 (two-way ANOVA following with Fisher’s LSD *post hoc* test).

In order to further understand whether PD-1 ligation and AHR activation act synergistically to suppress the IgM response, CD5^+/-^ B cells were treated with PDL2 and TCDD in combination followed by quantification of the IgM response on day 7. PDL2 treatment, either on day 0 or 3, did not further suppress the IgM response in AHR activated CD5^+^ B cells (**Figures 7A, B**). CD5^-^ B cells were refractory to AHR, or PD-1 activation as determined by the IgM response (**Figures 7A, B**).

### PD-1 Blockade With α-PD-1 Antibody Prevented the AHR-Mediated Suppression of the IgM Response in CD5^+^ ILBs

**Figure 8:**
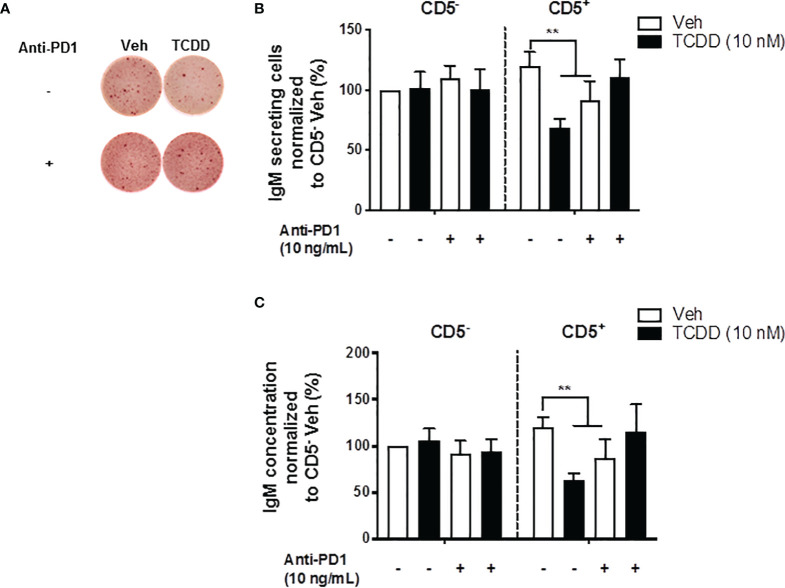
PD-1 antibody blockade prevented TCDD-mediated suppression of the IgM response in human CD5^+^ ILBs. Human CD5+/- B cells were isolated, activated, and treated with Veh or TCDD as described previously. To determine the contribution of PD-1 to TCDD-mediated IgM suppression, 10 ng/mL anti-PD-1 blocking antibody (S228P) was added to CD5^+/-^ B cell cultures. Following 7 days of culture, cells and culture supernatants were collected and assessed for IgM secretion. **(A)** Representative ELIspot wells showing the number of IgM secreting cells within CD5^+/-^ populations with or without anti-PD-1 treatment; **(B)** Averaged number of IgM secreting cells; and **(C)** Averaged IgM concentration from CD5^+/-^ culture supernatants. Determinations were made using B cells from 7 human donors (N = 7) across three independent experiments. Data presented in the figure were normalized to CD5^-^ Veh without anti-PD-1 treatment. Significant differences are indicated by **p < 0.01 (two-way ANOVA following with Fisher’s LSD *post hoc* test).

In the current studies, PD-1 ligation significantly suppressed the IgM response observed in CD5^+^ ILBs (**Figures 6A, B**). Therefore, we hypothesized that the PD-1 was critical in the suppression of the IgM response with AHR activation in CD5^+^ ILBs. To test this hypothesis, anti-PD-1 blocking antibody was used to block the interactions of PD-1 and it’s ligands in CD5^+^ ILBs. CD5^+^ and CD5^-^ B cells were activated as previously described and treated with either vehicle or 10 nM TCDD. In order to ensure the complete blockade of PD-1/PD-ligand interactions, anti-PD-1 blocking antibody was added on day 3 post activation, during which cells could upregulate PD-1 during day 1 to 2 post activation as PD-1 is engaged on lymphocytes as a part of the normal cellular activation process. Cells were collected 7 days post activation and the IgM response was quantified with ELISPOT and ELISA. CD5^+^ and CD5^-^ B cells both responded to activation by secreting IgM (**Figures 8A–C**). TCDD treated CD5^+^ B cells secreted ~50% less IgM compared to vehicle control (**Figures 8B, C**). Interestingly, PD-1 blockade also significantly reduced IgM secretion in CD5^+^ B cells in the absence of TCDD treatment compared to no antibody controls (**Figures 8B, C**). Despite this, when CD5^+^ B cells were treated with PD-1 blocking antibody treatment and TCDD, anti-PD-1 treatment prevented the TCDD-mediated IgM suppression in CD5^+^ ILBs as there was no difference between IgM secretion from anti-PD-1 plus TCDD co-treated CD5^+^ B cells and vehicle treatment alone (**Figures 8A–C**). Similar to our previous observation, CD5^-^ B cells were refractory to both TCDD and anti-PD-1 treatment (**Figures 8B, C**). Together these data directly demonstrate a role for PD-1 in the TCDD-mediated suppression of IgM secretion from CD5^+^ B cells. Further, these data also support a role for PD-1 engagement during lymphocyte activation for promoting optimal IgM release as PD-1 blockade alone modestly reduced IgM levels.

### Antibody Blockade of PDL1 and PDL2 Restores the Secretion of IgM in TCDD- Treated CD5^+^ Innate-Like B Cells

As we have previously shown that blocking PD-1/PD-1 ligand interactions restored the IgM response of CD5^+^ B cells treated with TCDD, we subsequently wanted to determine the relative contribution of blocking PD-1 ligands directly. As mentioned prior, PD-1 has two main ligands, PDL1 and PDL2, with PDL2 being of higher affinity. Blocking antibodies against PDL1 and PDL2 were employed to block their respective engagement with PD-1. As with the previous study, CD5^+^ and CD5^-^ B cells were isolated, activated and treated with TCDD as described. At the initiation of the culture period, 1 μg/mL of anti-PDL1, anti-PDL2, or both were added to the cultures. The rationale for the addition of the ligands at the initiation of the culture as compared to day 3 as with anti-PD-1 studies was to attempt to limit the negative effects on the IgM response. Addition of anti-PD-1 antibody alone reduced IgM secretion, which could be due to PD-1 interactions during the first 48 hours of activation.

**Figure 9:**
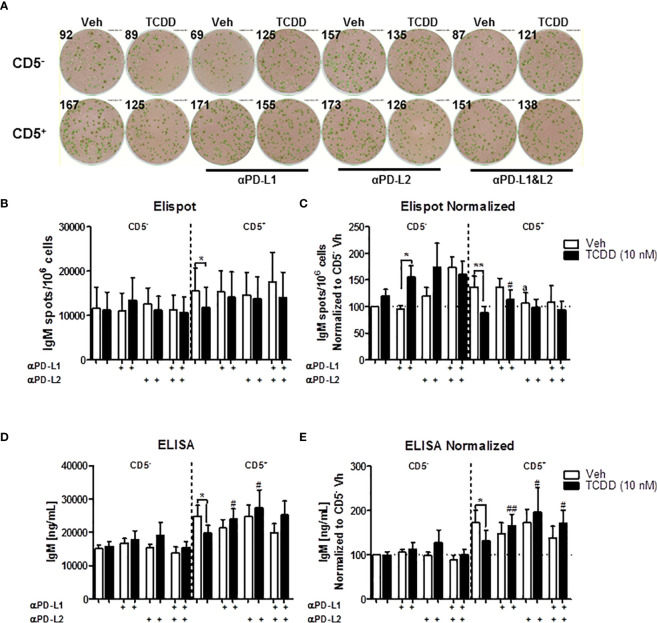
Antibody blockade of PD-L1 or PD-L2 promotes IgM secretion by TCDD-treated CD5^+^ B cells. To determine the contribution of PD-1 ligands to the TCDD-mediated suppression of IgM secretion, we leveraged PD-1 ligand antibody blockade. Briefly, human CD5^+/-^ B cells were isolated, activated, and treated (Veh or TCDD) as described. At the initiation of culture, 1 μg/mL of either αPD-L1 or αPD-L2 was added and cells were cultured for 7 days. Following the culture period, cells and culture supernatants were collected for IgM enumeration. Representative ELIspot wells from a single donor are shown in **(A)**. Averaged raw numbers of IgM secreting cells are shown in **(B)** and normalized data in (**C)**. Averaged IgM concentrations from culture supernatants are shown in **(D)** and normalized data are shown in (**E)**. Determinations were made using B cells from 8 human donors (N = 8) across three independent experiments. Data presented in the figure were normalized to CD5^-^ Veh without anti-PD ligand treatments. Significant differences between Veh and TCDD are indicated by *p < 0.05 and **p < 0.01. Significant differences between TCDD (no ligand blockade) and indicated TCDD-treated groups are indicated by ^#^ *p* < 0.05 and ^##^ *p* < 0.01. Significance was determined using a two-way ANOVA followed with a Tukey’s posttest. Significant differences between Veh and Veh treated with PD ligand are indicated by 'a' p < 0.05.

Similar as before, we observed a greater IgM response in CD5^+^ B cells compared to CD5^-^ B cells, which was suppressed with TCDD treatment (**Figures 9A–E**). When anti-PDL1 blocking antibody was added to culture, There was no significant effect on the IgM response in the absence of TCDD treatment (**Figures 9A–E**). However, in TCDD treated CD5^+^ B cells, blockade of PDL1 reversed TCDD-mediated suppression of the IgM response as there was no difference in the number of IgM secreting CD5^+^ B cells or concentration of IgM secreted when compared to control responses (**Figures 9C–E**). Contrary to blockade of PD-1, anti-PDL1 treatment in TCDD treated CD5^-^ B cells also enhanced the number of IgM secreting cells but did not affect IgM concentration in culture supernatants (**Figures 9C, E**). Unlike PDL1 blockade, administration of anti-PDL2 antibody did negatively affect the number of IgM secreting CD5^+^ B cells as evidenced by a ~25% reduction (**Figure 9C**). Further, the number of IgM spots in anti-PDL2 and TCDD treated CD5^+^ B cells were unchanged (**Figure 9C**). However, anti-PDL2 antibody treatment had no effect on the accumulation of IgM in culture supernatants as we did not observe a decrease with anti-PDL2 treatment alone. When PDL2 was blocked in TCDD-treated CD5^+^ B cells, we again observed IgM concentrations similar to control responses suggesting PDL2 blockade restores IgM secretion in TCDD treated CD5^+^ B cells (**Figures 9D, E**). Blocking both PDL1 and PDL2 did not affect the number of IgM secreting cells and also did not reverse the effects of treatment with TCDD (**Figure 9C**); however, blocking both ligands did restore IgM secretion in TCDD-treated CD5^+^ B cells (**Figure 9E**). Taken together, these data demonstrate that antibody blockade of either PDL1 or PDL2 can restore IgM secretion in TCDD-treated CD5^+^ B cells but only the blockade of PDL1 had an effect on the number of IgM secreting cells. Further, these data putatively suggest that the effects of PD-1 engagement on IgM secretion may strongly affect the number of IgM secreting cells as opposed to the amount of IgM they secrete. This is due to the observed differences between ELISPOT and ELISA measurements of the IgM response.

### IFNγ Treatment Reversed the AHR-Mediated Suppression of the IgM Response Through a Decrease of LCK in CD5^+^ ILBs

**Figure 10:**
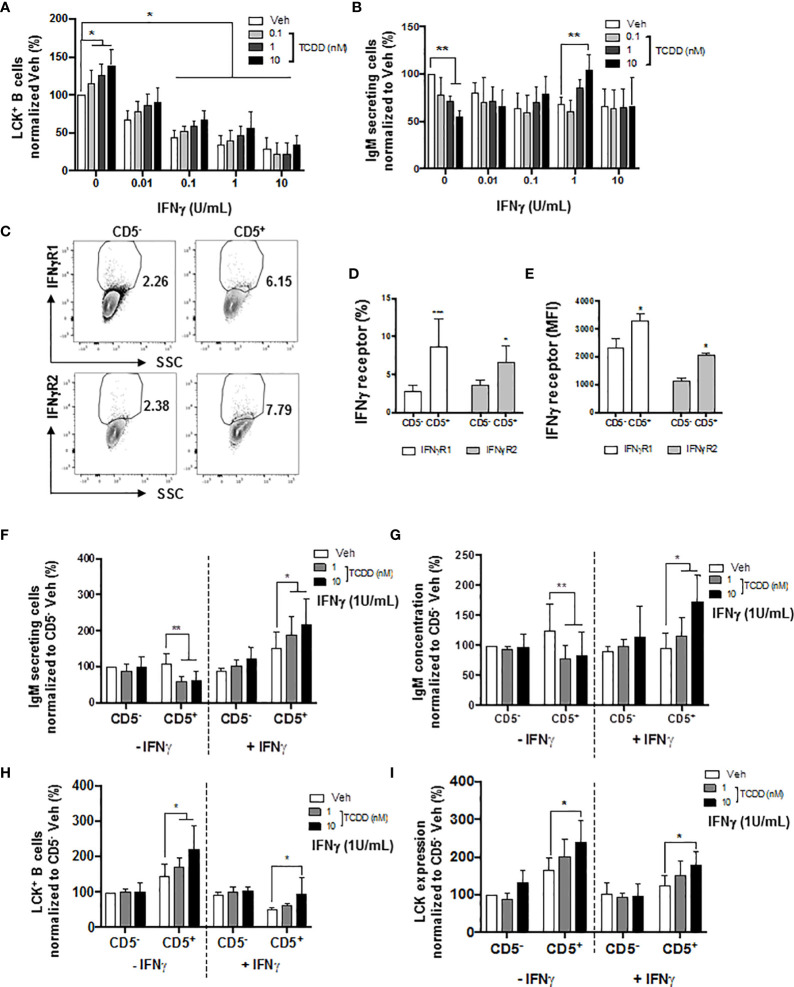
IFNγ treatment reversed the AHR-mediated suppression of the IgM response through a decrease of LCK in CD5^+^ ILBs. Human CD5^+/-^ B cells were activated/treated with soluble PDL1 or PDL2 on day 0 and cultured for 7 days as described previously. In addition, IFNγ treatment (1 U/mL) was provided on day 0. **(A)** Averaged frequency of LCK^+^ total B cells; **(B)** Averaged number of IgM secreting cells in CD19^+^ B cells; **(C)** Representative flow cytometry plots of IFNγR1^+^ and IFNγR2^+^ cells; **(D)** Averaged frequency of IFNγR1^+^ and IFNγR2^+^ cells; **(E)** Averaged MFI of IFNγR1 and IFNγR2 within CD5^+/-^ populations; **(F)** Averaged number of IgM secreting cells; and **(G)** Averaged IgM concentration within CD5^+/-^ culture supernatants treated with IFNγ; **(H)** Averaged percentage of LCK^+^ cells and; **(I)** averaged LCK MFI within CD5^+/-^ populations treated with IFNγ. Determinations were made using B cells from 6 human donors (N = 6) from two independent experiments. For **(A, B)**, data presented in the figure were normalized to the Veh group without IFNγ treatment. For F-I, data presented were normalized to the CD5^-^ Veh without IFNγ treatment. Significant differences are indicated by *p < 0.05, **p < 0.01 and ***p < 0.001 (two-way ANOVA following with Fisher’s LSD *post hoc* test).

A certain level of LCK is required in order to achieve optimal IgM production (52). Additionally, Blevins et al. have demonstrated that IFNγ treatment reversed AHR-mediated IgM suppression in human B cells *via* the modulation of STAT3 (54). Therefore, in the current studies, IFNγ was also employed as a molecular probe to further understand the role of AHR activation and LCK in the IgM suppression of CD5^+^ ILBs. First and foremost, a matrix study with increasing concentrations of both IFNγ and TCDD was conducted to investigate the interplay between LCK and IFNγ. Interestingly, with increased concentrations of IFNγ, the percentage of LCK positive cells decreased in a concentration-dependent manner, with significant suppression from 0.1 to 10 U/mL of IFNγ (**Figure 10A**). Activation of AHR continued to increase the percentage of LCK positive cells; however, the AHR-mediated increase was remarkably lower with IFNγ treatment compared to IFNγ non-treated B cells (**Figure 10A**). IFNγ treatment also reversed the AHR-mediated IgM suppression (**Figure 10B**). Combining the observations from **Figures 10A, B**, it is likely that the reversal of AHR-mediated suppression of the IgM response by IFNγ is by reducing LCK in human total B cells. Additionally, our studies showed that CD5^+^ ILBs were particularly sensitive to AHR activation (**Figures 1G, H**). Therefore, the level of IFNγ receptors was quantified in both CD5^+^ ILBs and CD5^-^ B cells. Interestingly, the level of IFNγ receptors (IFNγR1 and IFNγR2) was significantly higher in CD5^+^ ILBs compared to CD5^-^ B cells (**Figures 10D**, **E**). The high expression of IFNγ receptors on CD5^+^ ILBs suggests that this subset of B cells could be more responsive to IFNγ. Therefore, treatment with TCDD (10 nM) in combination with IFNγ (1U/mL) was used to determine if the percentage of LCK positive cells changed in relationship to the effects of TCDD and IFNγ on the IgM response in CD5^+^ ILBs. Similar to the observation in total B cells (**Figures 10A, B**); IFNγ treatment blocked AHR-mediated IgM suppression in CD5^+^ ILBs (**Figures 10F, G**). Interestingly, the percent positive of LCK significantly decreased with IFNγ treatment in CD5^+^ ILBs (**Figures 10H, I**) similar to that previously observed in total B cells (**Figure 10A**). Also, IFNγ treatment did not affect either the level of LCK or IgM response in CD5^−^ B cells (**Figure 10**).

### IFNγ Treatment Reversed the PD1-Mediated Suppression of IgM Secretion by CD5^+^ ILBs

**Figure 11:**
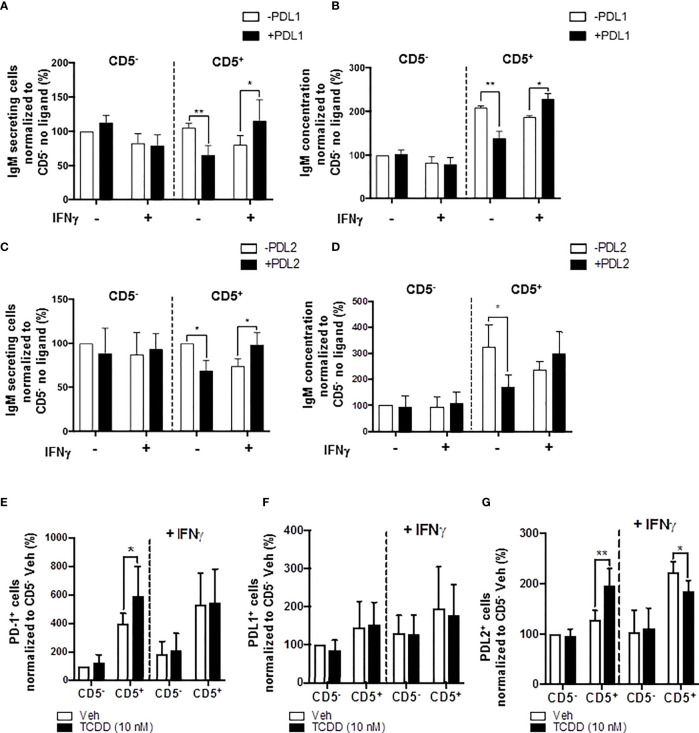
IFNγ treatment blocks PD-1-mediated IgM suppression in CD5^+^ ILBs. Human CD5^+/-^ B cells were activated, treated with soluble PDL1 or PDL2 on day 0 and cultured for 7 days as previously described. In addition, IFNγ treatment (1 U/mL) was provided on day 0. **(A)** Average number of IgM secreting cells; **(B)** Average IgM concentration from culture supernatants treated with soluble PDL1 within CD5^+/-^ cultures in the presence of IFNγ treatment; **(C)** Averaged number of IgM secreting cells; **(D)** Average IgM concentration from culture supernatants treated with soluble PDL1 within CD5^+/-^ cultures in the presence of IFNγ treatment; **(E)** Average frequency of PD-1^+^ cells; **(F)** Average frequency of PDL1^+^ cells; and **(G)** Average frequency of PDL2^+^ in CD5^+/-^ populations with or without IFNγ treatment. Determinations were made using B cells from 6 human donors (N = 6) across two independent experiments. For **(A–D)**, results were normalized to CD5^-^ B cells without soluble PD-1 ligand treatment. For **(E–G)**, results were normalized to the Veh control group in CD5^−^ B cells. Significant differences are indicated by *p < 0.05 and **p < 0.01 (two-way ANOVA following with Fisher’s LSD *post hoc* test).

In the current study, we show results suggesting that IFNγ treatment reversed the AHR-mediated IgM suppression through a decrease in LCK in CD5^+^ ILBs (**Figure 10**). In addition, previous studies have demonstrated that IFNγ signaling can regulate the expression of PD-1 and PDL1 (56, 57). Additionally, PD-1 signaling suppressed the IgM response in CD5^+^ ILBs (**Figure 6**). Therefore, to further understand the role of PD-1 signaling in CD5^+^ ILBs, IFNγ (1 U/mL) was used to treat CD5^+^ ILBs in the presence of sPDL1 (1 μg/mL) or sPDL2 (50 ng/mL). IFNγ treatment reversed the PD-1-mediated IgM suppression in CD5^+^ ILBs (**Figures 11A–D**). Curiously, despite inhibiting PD-1-mediated suppression of IgM secretion, IFNγ treatment enhanced the frequency of CD5^+^ ILB positive for PD-1 in the absence of TCDD treatment suggesting that IFNγ regulates PD-1 signaling (**Figure 11E**). Furthermore, the frequency of CD5^+^ ILBs expressing PDL1 appeared to be largely unaffected by either TCDD-mediated AHR activation or IFNγ treatment (**Figure 11F**). Interestingly, IFNγ treatment increased the percentage of PDL2 positive cells within CD5^+^ ILBs treated with Veh alone (**Figure 11G**). In fact, the frequency of CD5^+^ ILB treated with only IFNγ was higher than those treated with both IFNγ and TCDD (**Figure 11G**). Considering that TCDD-treated CD5^+^ B cells had similar frequencies of PDL2 positivity as CD5^+^ B cells treated with both TCDD and IFNγ, our results strongly suggest that IFNγ negatively regulates PD-1 signaling in human CD5^+^ ILB.

## Discussion

AHR activation has consistently been demonstrated to suppress antibody responses in most experimental models tested, including humans. However, the mechanism remains poorly understood in humans in comparison to rodent models. Studies have shown that AHR activation increases BCL-6 and SHP-1, which corresponds with impaired human B cell activation (63, 64). More recently, studies have indicated that AHR activation also increases LCK levels and the percentage of LCK positive B cells while impairing IgM response in the total CD19^+^ human B cell pool (52). The finding that AHR activation led to an upregulation of LCK was intriguing as LCK is not commonly associated with B cell activation and differentiation. In fact, LYN is the predominant SRC kinase in B cells due to its involvement in B cell receptor (BCR) signaling. Therefore, the ability of AHR activation to upregulate LCK in human B cells was both unexpected and curious. However, a recent publication by Till and coworkers demonstrated that CD5^+^ human B cells, which comprise approximately 5%-25% of circulating B cells, express high levels of LCK (27). CD5^+^ B cells, while being heterogenous, have generally been characterized as innate-like and shown to produce copious amounts of IgM (1). These findings were particularly interesting in light of previous studies which demonstrated that AHR-mediated impairment of IgM responses usually does not exceed 50% when compared to the vehicle control group (65, 66). This observation suggested that different populations of B cells might have differential sensitivity to AHR activation. In fact, Blevins et al. recently demonstrated high basal levels of *AHR* mRNA in human CD5^+^ ILBs in comparison to total CD19^+^ or CD5^-^ B cells (17). Furthermore, human CD5^+^ ILBs expressed higher levels of *PD-1*, *PDL1* and *PDL2* mRNA as well as significantly more PD-1 and PDL2 protein on the surface of CD5^+^ ILB compared to CD5^-^ B cells (17). Consequently, AHR activation significantly and preferentially suppressed IgM responses in CD5^+^ ILBs, but not in CD5^-^ B cells (17).

Here, we extend these prior observations by further examining the role of LCK and PD-1 in the context of AHR activation on CD5^+^ ILB function. However, as with our previous study (17), the goal of the current study was not to positively identify human B1 B cells directly. Many groups have purported to have identified the human B1 surrogate and many of these classification schemes include CD5. In contrast to CD5^+^ ILB, many groups also denote human ‘B1 B cells’ as CD27^+^ (25), which is still controversial as well. For example, Griffin and colleagues reported that human CD19^+^CD27^+^CD20^+^CD43^+^CD70^-^ cord blood cells were a human ‘B1’ surrogate. They showed that they spontaneously secreted IgM in the absence of stimulation as determined by a lack of CD69 expression. By contrast, Blevins and coworkers also demonstrated previously that human circulating CD19^+^CD5^+^CD27^-^ ILB also secrete high levels of IgM in the absence of stimulation (17). In fact, a report by Seifret and colleagues compared CD5^+^CD27^-^ and CD5^+^CD27^+^ B cells from healthy donors and concluded that the expression of CD27 by CD19^+^CD5^+^ B cells denoted cells which had previously undergone a germinal center reaction based on gene signatures (67) but did not conclude that CD27 expression denoted a ‘different’ cell type altogether. Despite these differences we have previously reported remarkable similarity in the profiles of murine B1 B cells and circulating human CD5^+^ ILBs in regard to expression of PD-1 (61, 68, 69), AHR (70, 71), and LCK (11, 72) which we have expanded upon currently (17).

In the current studies, CD5^+^ B cells and LCK^+^ B cells in humans were highly correlated, demonstrating that this subpopulation of B cells express high levels of intracellular LCK. After magnetic enrichment, the basal level of LCK was also significantly higher in CD5^+^ ILBs compared to CD5^-^ B cells. The percentage of LCK^+^ CD5^+^ ILBs further increased with AHR activation over a 7-day culture period. This observation is in agreement with previous findings showing that high LCK expressing cells are largely CD5^+^ B cells in peripheral blood (11, 27). In addition, AHR activation significantly suppresses IgM response in only CD5^+^ ILBs but not in CD5^-^ B cells following 7-day culture. These findings show that CD5^+^ ILBs are particularly sensitive to AHR activation. Furthermore, AHR antagonist treatment attenuated LCK upregulation and suppression of the IgM response in CD5^+^ ILBs. This observation indicates that AHR activation is crucial in eliciting an increase in LCK and suppression of the IgM response in CD5^+^ ILBs. Previously, we also demonstrated that LCK inhibitor treatment could reverse AHR-mediated IgM suppression in human B cells (52). Similarly, in the current studies, LCK inhibitor treatment reversed AHR-mediated IgM suppression in CD5^+^ ILBs, demonstrating that LCK activity is critically involved in the IgM response by CD5^+^ ILBs. Moreover, LCK inhibitor treatment alone is also inhibitory, suggesting an optimal level of LCK is required for the IgM response. In our previous study, LCK protein levels were non-detectable from day 0 to 2 in our culture system until day 3 post B cell activation, which is in contrast with our current observation. One possible explanation is that our previous studies were conducted in total CD19^+^ B cells which could putatively lower the sensitivity of detection of LCK in a small subpopulation (CD5^+^ ILBs) within the total B cell pool. Physical separation of CD5^+^ ILBs from total B cells enhanced the detection sensitivity to observe the high level of LCK in this small subset of B cells. Interestingly, epidemiological studies have shown a positive correlation between AHR activation and Non-Hodgkin’s Lymphoma (NHL). High expression of LCK has been reported in CLL patients, a form of NHL. It is tempting to speculate that the increase of LCK in CD5^+^ ILBs could potentially contribute to the development of CLL in humans. In addition, due to the nature of LCK as a tyrosine kinase, it is likely that LCK could phosphorylate downstream targets involved in inhibitory responses, which at supraoptimal levels results in the down-regulation of the IgM response by CD5^+^ ILBs. Further investigation is required to identify downstream targets of LCK in CD5^+^ ILBs.

PD-1, an immune check-point inhibitor, and its ligands (PDL1 and PDL2) represent a hallmark of immune regulation. Recent studies have demonstrated that CD5^+^ ILBs expressed high level of PD-1 (17). In addition, the high level of PD-1 expression by CD5^+^ ILBs also suggests that they may be particularly sensitive to PD-1-mediated regulation (13). However, little is known about PD-1 signaling in the regulation of IgM responses (60). It has been shown that LCK can phosphorylate the ITSM and ITIM domains of PD-1 (73). Therefore, studies were conducted to further understand the role of LCK and PD-1 in the context of AHR activation in CD5^+^ ILBs. Recently, we reported that the level of PD-1 increases significantly with AHR activation (17). Further, we report here that PDL2, but not PDL1 is also significantly upregulated by TCDD treatment. In contrast to our findings, it has been reported that AHR-activation can enhance the expression of PDL1 (74, 75). While we did not see effects on PDL1 with TCDD-mediated AHR activation, this could be due in part to the AHR ligand utilized. For example, one of the reports that AHR activation enhanced PDL1 expression utilized BαP from cigarette smoke to activate AHR (74). The other published example demonstrated that kynurine-mediated activation in cancer cells enhanced PDL1 expression (75). These differences are likely due to the fact that AHR is differentially modulated depending on the specific ligand that activates it resulting in highly context/tissue/cell type dependent effects of AHR activation (76).

The observation that TCDD-mediated AHR activation enhances PDL2 protein expression further suggests that AHR-mediated upregulation of PD-1 can potentially activate inhibitory signals in CD5^+^ ILBs. In the current study, recombinant soluble PD-1 ligands (PDL1 and PDL2) were used to mimic ligand interactions with PD-1. Interestingly, treatment with soluble PDL1, PDL2 or a combination of both significantly suppressed the IgM response in CD5^+^ ILBs. In addition, we show that antibody blockade of PD-1 prevents AHR-mediated suppression of the IgM response in CD5^+^ ILBs; further suggesting that PD-1 signaling plays a critical role in the regulation of the IgM response in CD5^+^ ILBs. Indeed, we extended this finding to demonstrate that the antibody blockade of both PDL1 and PDL2 restored IgM secretion in TCDD-treated CD5^+^ B cells. To our knowledge, this is the first report of PD-1 engagement regulating the IgM response in human B cells. Despite a dearth of human-specific data in regard to PD-1 regulating antibody responses, it is noteworthy that a study by McKay and coworkers reported similar findings in mice (60). They found that blocking PD-1 or selectively blocking PDL2 on antigen presenting cells enhanced murine antibody titers to infection with *Streptococcus pneumoniae* (60). A key difference in our study was that we observed a putative role for PDL1 in also regulating human IgM secretion. However, these variations could be due to species-specific differences between mice and human. Further, McKay and coworkers made their observations in both genetic knockouts as well as an infection model. More work in human CD5^+^ B cells will be necessary to assess the contributions of PDL1 and PDL2 in regulating humoral responses.

AHR activation also increased LCK expression in CD5^+^ ILBs and LCK is known to phosphorylate PD-1 (62). To further understand the interplay between PD-1 and LCK, a specific LCK inhibitor (RK24466) was used in combination with PDL2 treatment in CD5^+^ ILBs. LCK inhibitor treatment prevented PD1-mediated IgM suppression, indicating that the inhibition of LCK results in the failure of PD-1 engagement to suppress IgM secretion. This observation further suggests that LCK plays a critical role in PD-1 signaling and the activity of LCK govern the IgM response in CD5^+^ ILBs. While it is tempting to speculate that TCDD-treatment may synergize with PD-1 signaling to result in enhanced immune suppression, this is likely not the case. For example, treatment with sPDL2 and TCDD did not further suppress the IgM response compared to CD5^+^ ILBs treated with TCDD alone, where the suppression of the IgM response remained around 50% compared to Veh control. This suggests that there is no synergistic effect between AHR and PD-1 activation. An alternate possibility is that it may be impossible to achieve 100% IgM suppression in CD5^+^ ILBs given the constitutive nature of their secretion of IgM. Lastly, ILBs are a relatively heterogeneous population with different sub-populations. This gives rise to the potential scenario where a specific subset of CD5^+^ ILB may be exhibiting differential sensitivity to AHR activation. This could manifest as a direct effect of AHR-activation in IgM secreting cells or as an indirect effect by eliciting inhibitory signals from a non-antibody secreting population such as B_regulatory_ cells (B_reg_). It is noteworthy that some B_reg_ populations are overrepresented in CD5^+^ ILB, such as CD9^+^ B_regs_ (77). Exploration of downstream PD-1 signaling could help identify other targets that are involved in the AHR-mediated impairment of the IgM response in CD5^+^ ILBs as PD-1 is one of the many inhibitory receptors (CTLA-4, TIM-3, CD22, BTLA and LAG-3) expressed by CD5^+^ ILBs (78). Likewise, there may be other ligands for PD-1 beyond those described in the literature. One example is B7-H3 which has recently been described in promoting immune suppression in the tumor microenvironment (79, 80). Further investigation of these inhibitory receptors and additional PD-ligands are required to better understand their role of AHR activation in CD5^+^ ILBs.

Finally, IFNγ has been suggested to have suppressive effects on humoral immunity. However, previous studies have been conducted using high IFNγ concentration (≤1000U) (81). In the presence of low IFNγ concentrations, IFNγ treatment can enhance humoral immunity and reverse the AHR-mediated IgM suppression (53). Furthermore, IFNγ treatment can regulate expression of PD-1 and PDL1 (57). Therefore, in the current studies, IFNγ was used as a molecular probe to further elucidate the role of LCK and PD-1 in CD5^+^ ILBs. First and foremost, a significant decrease in the percent of LCK^+^ B cells was observed with IFNγ treatment. This finding is particularly interesting since the decrease of LCK with IFNγ treatment correlates with the restoration of the IgM response. To our knowledge, there are no reports of IFNγ negatively regulating LCK in such a manner. However, given the rapidity in which IFNγ administration drastically reduced LCK protein levels, it is tempting to speculate as to the mechanism involved. There are published reports of IFNγ signaling resulting in the generation of immune-specific proteasomes which can degrade targeted proteins (82). It is likely that, by modulating LCK, IFNγ treatment is able to reverse the AHR-mediated suppression of the IgM response in B cells. Furthermore, IFNγ receptor expression is significantly higher in the CD5^+^ ILBs compared to CD5^-^ B cells, indicating that CD5^+^ ILBs are more sensitive toward IFNγ treatment compared to CD5^-^ B cells. Moreover, IFNγ treatment reversed PD-1-mediated suppression of the IgM response in CD5^+^ but not CD5^-^ ILBs. Overall, IFNγ treatment enhanced the frequency of expression of PD-1 and PDL2 in CD5^+^ ILBs. This is in contrast to reports that IFNγ signaling regulates PDL1 expression (55). These observations strongly suggest IFNγ treatment regulates PD-1 signaling despite increasing the frequency of protein expression. Taken together, the decrease of LCK with IFNγ treatment could potentially lead to decreased phosphorylation of the PD-1 signaling domain and an inability to send negative signals to the cell. Further investigations are required to fully understand the mechanism of IFNγ interaction in the PD-1 signaling in CD5^+^ ILBs.

**Figure 12:**
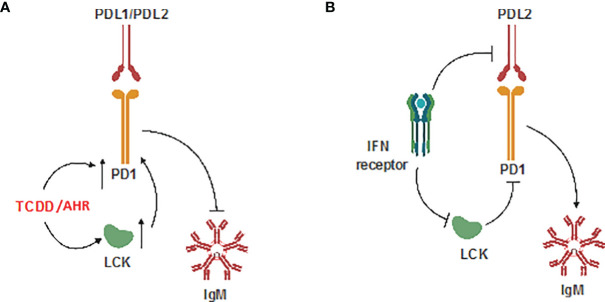
Schematic of potential mechanism. **(A)** Effect of TCDD-mediated AHR activation; and **(B)** Effect of IFNγ on CD5^+^ ILBs.

These results provide, for the first time, a detailed mechanism by which AHR activation results in the upregulation of LCK and PD-1 which results in suppression of the IgM response in human CD5^+^ ILBs. Additionally, antibody blockade of PD-1 or its ligands restored IgM secretion in TCDD-treated CD5^+^ B cells suggesting, for the first time, a direct role for PD-1 in TCDD-mediated immunotoxicity. We also report that LCK is actively required to facilitate the inhibition of IgM secretion by PD-1 engagement as functionally inactivating LCK prevented soluble PD-ligands from suppressing IgM secretion. Lastly, we demonstrate the capacity of IFNγ administration in promoting IgM secretion in CD5^+^ B cells treated with either TCDD or soluble ligands of PD-1 which corresponds to decreased LCK protein expression in response to IFNγ. While these findings, which have been summarized in **Figure 12**, provide the most direct putative mechanism for AHR-mediated suppression of IgM secretion in human CD5^+^ B cells, future studies will be necessary to identify the specific targets of AHR activation within CD5^+^ ILB.

## Data Availability Statement

The original contributions presented in the study are included in the article/supplementary material. Further inquiries can be directed to the corresponding author.

## Ethics Statement

Ethical review and approval was not required for the study on human participants in accordance with the local legislation and institutional requirements. Written informed consent for participation was not required for this study in accordance with the national legislation and the institutional requirements.

## Author Contributions

JZ, LB, RC, and NK contributed to the development of the experimental design and interpretation of results. JZ and LB conducted the experiments. JZ and LB co-wrote the paper, with edits by RC, and NK. All authors contributed to the article and approved the submitted version.

## Funding

The work was supported by NIH grant P42 ES004911.

## Conflict of Interest

The authors declare that the research was conducted in the absence of any commercial or financial relationships that could be construed as a potential conflict of interest.

## Publisher’s Note

All claims expressed in this article are solely those of the authors and do not necessarily represent those of their affiliated organizations, or those of the publisher, the editors and the reviewers. Any product that may be evaluated in this article, or claim that may be made by its manufacturer, is not guaranteed or endorsed by the publisher.
